# Systematic review of randomised and observational evidence of effects of treatments of carotid stenosis to prevent stroke

**DOI:** 10.1186/1745-6215-16-S2-P164

**Published:** 2015-11-16

**Authors:** Kusal Lokuge, Alison Halliday, Richard Bulbulia, Alastair Gray, Borislava Mihaylova

**Affiliations:** 1 HERC, Nuffield Department of Population Health, University of Oxford, Oxford, Oxfordshire, UK; 2 Nuffield Department of Surgical Sciences, University of Oxford, Oxford, Oxfordshire, UK; 3 CTSU, Nuffield Department of Population Health, University of Oxford, Oxford, Oxfordshire, UK

## Background

The most effective treatment of carotid stenosis in different types of patients is currently uncertain. Procedural improvements might have reduced peri-procedural risks of both Carotid Endarterectomy (CEA) and Carotid Artery Stenting (CAS), while the increased use of effective medical therapies has reduced stroke risks.

## Purpose

To systematically review randomised and observational evidence of peri-procedural and long-term effects of CEA, CAS and Medical Treatment Alone (MTA) for individuals with substantial carotid stenosis. To discuss the contribution of observational evidence in informing implications of changing disease risks and procedural characteristics on best treatment strategies in categories of patients.

## Method

We reviewed Randomized Control Trials (RCTs) reporting peri-procedural and/or long-term risks of CEA vs MTA and CEA vs CAS. In addition, a systematic review identified observational studies studying peri-procedural and long-term effects of CEA or CAS that recruited more than 500 participants and followed them for at least 30 days. Where available, results were tabulated under sub-group classifications including gender, age and symptomatic status.

## Results

The meta-analysis from RCTs suggests that compared to CAS, CEA is associated with lower peri-procedural [OR:0.58(0.46-0.75)] risks of Stroke/Death for symptomatic patients, but the evidence is inconclusive in asymptomatic patients [OR:0.53(0.22-1.27)]. In the long-term CEA is reported to reduce stroke risk in symptomatic and mixed populations [OR:0.68(0.58-0.80)]. RCTs comparing CEA versus MTA suggests that in the long-run asymptomatic CEA patients have lower stroke risks [OR:0.39(0.28-0.53)]. More than 50 observational studies reporting results from CEA and CAS patient cohorts have been identified and are being reviewed.
